# Lung‐specific exosomes for co‐delivery of CD47 blockade and cisplatin for the treatment of non–small cell lung cancer

**DOI:** 10.1111/1759-7714.14606

**Published:** 2022-08-27

**Authors:** Zhilei Cui, Zhengshang Ruan, Junxiang Zeng, Jinyuan Sun, Wenjing Ye, Weiguo Xu, Xuejun Guo, Linlin Zhang, Lin Song

**Affiliations:** ^1^ Department of Respiratory Medicine XinHua Hospital Affiliated to Shanghai Jiao Tong University School of Medicine Shanghai China; ^2^ Department of Infectious Disease XinHua Hospital Affiliated to Shanghai Jiao Tong University School of Medicine Shanghai China; ^3^ Department of Laboratory Medicine XinHua Hospital Affiliated to Shanghai Jiao Tong University School of Medicine Shanghai China; ^4^ Department of Nuclear Medicine Xinhua Hospital Affiliated to Shanghai Jiao Tong University School of Medicine Shanghai China

**Keywords:** anticancer, CaCE, CDDP, exosome delivery

## Abstract

A cluster of differentiation 47 (CD47) and immune‐modulatory protein for myeloid cells has been implicated in cisplatin (CDDP) resistance. Exosome delivery of drugs has shown great potential for targeted drug delivery in the treatment of various diseases. In the current study, we explored the approach of co‐delivering CDDP and CD47 antibody with MDA‐MB‐231 cell‐derived exosome 231‐exo (CaCE) and assessed the phagocytosis activity of bone marrow flow cytometry derived macrophages (BMDM) against co‐cultured A549 cells. CD8^+^ T‐cell proliferation was examined with flow cytometry analysis. In vivo, we used the Lewis lung carcinoma (LLC) tumor‐bearing mouse model and assessed survival rate, tumor weight, phagocytosis, and T‐cell proliferation, as well as cytokine levels in tumors analyzed by enzyme‐linked immunoassay (ELISA). Although co‐administration of CDDP with anti‐CD47 (CDDP and aCD47) showed a significant antitumor effect, CaCE had an even more dramatic anticancer effect in survival rate and tumor weight. We observed increased phagocytosis activity selectively against lung tumor cells in vivo and in vitro with exosome CaCE treatment. CaCE treatment also increased T‐cell proliferation compared to the vehicle treatment and co‐administration groups. Furthermore, immunostimulatory interleukin (IL)‐12p and interferon (IFN)‐γ were increased, whereas transforming growth factor β (TGF‐β) were decreased, indicating the improved CDDP anticancer effect is related to a tumor microenvironmental change. Our study demonstrates a dramatically improved anticancer effect of CDDP when administered by exosome co‐delivery with anti‐CD47.

## Graphical abstract

An approach of co‐delivering cisplatin (CDDP) and cluster of differentiation 47 (CD47) antibody with MDA‐MB‐231 cell‐derived exosome 231‐exo (CaCE) was developed. It was demonstrated that a dramatically improved anticancer effect of CDDP when administered by exosome co‐delivery with anti‐CD47.

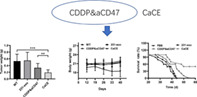

## INTRODUCTION

Lung cancer is one of the leading causes of death with a staggering 2.09 million increase in diagnosis each year.^1^, ^2^ Therapeutic options include chemotherapy, immunotherapy, and targeted therapy. The platinum‐based drugs, alone or in combination with other medications, have been the primary treatment choice in viable situations.^3^, ^4^ Platinum applied its anticancer effect through DNA adducts creation of DNA distortion, which activates the nucleotide excision repair (NER) pathway leading to cell death in rapidly dividing cells such as cancers.^5^ Platinum resistance in cancers results either from reduced drug accumulation or reduced cell response in tumor cells and leads to poor clinical outcomes in patients.^6^ Tumor microenvironment changes are an important factor for drug resistance in chemotherapy,^7^ including changes in protumor and antitumor cytokines.^8^ Another challenge of traditional chemotherapy, including cisplatin, is systemic toxicity because of lack of tissue specificity.^9^ Side effects on the kidneys, digestive tract, and other parts of the body that involve rapid cellular turnaround compromise the clinical outcome of cisplatin treatment.^10^, ^11^ Novel approaches that increase drug delivery efficiency and tissue specificity and reduce cisplatin resistance greatly benefit its clinical application.

The cluster of differentiation 47 (CD47) is universally expressed on normal cells, and tumor cells regulate phagocytosis and cytotoxic activity of myeloid cells through interaction with the signal‐regulatory protein α (SIRPα) receptor on myeloid lineage derived cells.^12^, ^13^ Our previous work found that CD47 blockade enhanced macrophage phagocytosis of tumor cells both in vivo and in vitro, enhancing the therapeutic effect of cisplatin on lung cancer in vitro and in vivo.^14^

Exosomes are bilipid‐layered vesicles ranging 30–150 nm in diameters secreted from cells loaded with various cargo materials.^15^ The exosome component varies with the tissue of origin and serves cellular communication functions.^16^ Exosomes have high biocompatibility and low immunogenicity compared to synthetic nanoparticle delivery systems and can efficiently deliver therapeutic agents, nucleic acid, or proteins.^17^, ^18^ Exosomes play an important role in cancer organotypic metastasis.^19^ Exosome membrane protein integrins contribute to tissue targeting of exosome,^20^ which provided a potential tissue targeted delivery of therapeutic cargo when properly engineered.^21^ This clinical potential of exosomes has been widely explored in recent years. 231‐Exo, a breast cancer cell line MDA‐MB‐231, derived exosomes efficiently delivered microRNA (miRNA) selectively into A549, a non–small cell lung cancer cell line. Integrin β4 overexpressed on 231‐exo interacts with surfactant protein C (SPC) on cancer cells and is specifically internalized by non–small cell lung cancer cells.^22^

In the current study, we designed 231‐exo‐based cisplatin and anti‐CD47 co‐delivery system and tested its anticancer effect in vivo and in vitro.

## MATERIALS AND METHODS

### Cell culture

MDA‐MB‐231 (HTB‐26), Lewis lung carcinoma (LLC; CLR1642), GFP Reporter Cell Line‐LLC (LLC‐GFP; CSC‐RR0525); A549 (CCL‐185), NIH‐3T3 (CRL‐1658), DC2.4 mouse dendritic cells (SCC142) lines were procured from the Cell Bank of the Chinese Academy of Sciences and were cultured at 5% CO_2_, 37°C. The cells were cultured in media supplemented with 10% fetal bovine serum (FBS) (HyClone) and 1% penicillin–streptomycin (Glenview). A549 cells in F‐12K medium (HyClone), human embryonic lung fibroblast (HELF) cells‐Dulbecco's Modified Eagle Medium (DMEM medium) (HyClone).

### Primary bone marrow‐derived macrophages culture

After sacrificing the mice by CO_2_ inhalation, the tibial bones were carefully dissected marrow cells were flushed from femurs and tibia of age‐matched mice (6–8 weeks) by perfusion with 2 mL of phosphate buffer saline (PBS), pH 7.4 (10‐010‐031; Fisher Scientific). The resulting cell suspension was further dissociated by pipetting and centrifuged at 300 × *g*. Bone marrow‐derived macrophages were differentiated by culturing bone marrow cells in DMEM supplemented with 20% FBS and CSF1 (50 ng/ml; 300‐25 Peprotech) for 4–6 days.

### Preparation and purification of empty exosomes

MDA‐MB‐231 cells were maintained in FBS‐free media for 48 h. The cultures were collected and subjected to a series of centrifugations: 300 × *g* for 10 min; 2000 × *g* for 10 min; 10 000 × *g* for 30 min. After each centrifugation step, the supernatants were transferred to the new tubes and pellets were discarded. Following centrifugations, PureExo Exosome Isolation kit was used for the exosomes isolation.

### Preparation of cisplatin anti‐CD47 antibody loaded exosomes

ExoFectin sRNA‐into‐Exosome Kit was used for the development of a combined cisplatinum payload. Briefly, 25 μL of TransExo buffer and 5 μL of TransExo reagent were mixed. Next, cisplatin (>99% purity, Boyuan Chemical Company) and CD47 antibody (Clone MIAP410, Bio X Cell) (in 25 μL TransExo buffer) was added into the mixture and incubated for 5 min at room temperature. Subsequently, 50 μg of exosomes were added into the solution and incubated overnight at 37°C. The free cisplatin and unconjugated CD47‐antibody were eliminated by PBS washing, and ultracentrifugation 50 000 × *g* for 6 h at 4°C supernatants were then discarded exosome pellet resuspended with PBS and measured for protein concentration.

### Real‐time high‐resolution particle detection

Nanoparticle tracking analysis (NTA) was used for the exosome counting, and NanoSight NS300 (Malvern Instruments) was used for size distribution and concentration (particles/mL) analysis.^23^

### Transmission electron microscopy

Exosome isolates were fixed in 2% paraformaldehyde, mounted on copper‐mesh grids (Electron Microscopy Sciences), and uranyl acetate at 2% was used for negative staining. JEOL 1230 transmission electron microscope (JEOL) was used for imaging. Image analysis was performed using a software package bundled with the microscope.

### Western blotting

Samples were lysed in radioimmunoprecipitation assay (RIPA) buffer, sonicated, and centrifuged 15 000 × *g* for 10 min at 4°C to remove cell debris. Total protein concentrations of supernatants were determined using a BCA kit (Thermo Fisher Scientific). Total proteins were loaded to sodium dodecyl sulfate–polyacrylamide gel electrophoresis (SDS‐PAGE), and the separated proteins were transferred to polyvinylidene fluoride (PVDF) membranes (FL00010, Millipore‐Sigma). PVDF membranes were then blocked for 1 h with 5% fat‐free milk at room temperature, before incubation with primary antibodies against CD9 (ab223052, 1;500, Abcam), CD63 (ab1318, 1;1000, Abcam), CD47 (ab214453, 1;1000, Abcam). GAPDH (MA5‐15738; 1:2000, Thermo Fisher Scientific) was used as the loading control. Membranes were then incubated with horse radish peroxidase (HRP)‐conjugated secondary antibodies for 1 h. Following an additional three TBS‐T washing cycles. All membranes were detected using enhanced chemiluminescence Pierce ECL Western Blotting Substrate (Thermo Fisher Scientific).

### RNA isolation and quantitative real time‐polymerase chain reaction

RNA was extracted by RNeasy Plus Mini Kit (Qiagen) the resulting isolated RNA was quantified using a NanoDrop One unit (Thermo Fisher Scientific). RNA was reverse‐transcribed to complementary DNA (cDNA) using Maxima H Minus cDNA Synthesis Master Mix (dsDNase, M1682; Thermo Fisher Scientific). Gene expression was analyzed using Power SYBR Green Master Mix (Thermo Fisher Scientific) with the following mouse‐specific primers: CD47‐F: GCTTCTGGACTTGGCCTCAT, CD47‐R: CCTCTGGTTGGAAGCGACAA; GAPDH was used as housekeeping gene (GAPDH‐F: GATCCCGCTAACATCAAATG, GAPDH‐R: GAGGGAGTTGTCATATTTCTC). Reactions were detected by QuantStudio 3 real‐time polymerase chain reaction (RT‐PCR) system (Applied Biosystems). The relative expression values of the target genes were normalized to GAPDH, and the fold change was calculated using the relative quantification (2^−ΔΔCT^) method.

### Animals

C57BL/6 mice, 6–8 weeks old, were purchased from Shanghai Laboratory Animal Center (Shanghai, China). All animal procedures were approved by the Ethical Committee of XinHua Hospital Affiliated to Shanghai Jiao Tong University School of Medicine.

C57BL/6 mice were subcutaneously injected with 10^6^ LLC or LLC‐GFP cells mixed in 50 μL matrigels/tumor for the LLC‐bearing mouse model. When the tumor tissues reached 50 mm^3^, the mice were injected through the tail vein with cisplatin (CDDP) at 2.5 mg/kg and aCD47 at 2.5 mg/kg, 231‐exo, 231‐exo loaded with CDDP (2.5 mg/kg) and aCD47 (2.5 mg/kg) or equal volume of PBS every 3 days up to day 12. After treatment, tumor volume was measured every 3 days until day 21. At the end, the mice were euthanized with cervical dislocation and the tumor tissues were collected for further assays.

### Survival experiment

C57BL/6 mice were inoculated with LLC cells subcutaneously and treated with CDDP (2.5 mg/kg) and aCD47 (2.5 mg/kg), 231‐exo, 231‐exo loaded with CDDP (2.5 mg/kg) plus aCD47 (2.5 mg/kg), or an equal volume of PBS for up to 9 days then the treatment was stopped, and animal observation was conducted for 60 days.

### Phagocytosis assay

Phagocytosis assay was performed as previously reported with.^24^ Briefly, LLC cells were incubated with fluorescent dye carboxyfluorescein succinimidyl ester (CFSE) at 5 μM at 37°C for 20 min. After washing away free CFSE, bone marrow flow cytometry derived macrophages (BMDMs) were mixed with LLC in the presence of CDDP and aCD7, 231‐exo, cell‐derived exosome 231‐exo (CaCE), or vehicle at a ratio of 1:1 for 48 h. The cells were then incubated with the F4/80‐PE‐Cy7 antibody (Clone BM8, BioLegend) for flow cytometry analysis, and the percentage of CFSE^+^, F4/80^+^ phagocytotic macrophages were quantified.

### Enzyme‐linked immunoassay

Tumor tissues were lysed and homogenized in RIPA buffer, levels of cytokines were determined using specific enzyme‐linked immunoassay (ELISAs), including transforming growth factor β (TGFβ) (DB100C; R&D systems), interleukin (IL)‐12p70 (M1270; R&D systems), and interferon (IFN)‐γ (MIF00; R&D systems). Optic density was detected using Synergy H1 (Biotek), the optical density (OD) was recorded at 450 nm with references wavelengths at 570 nm.

### Flow cytometry

293T, A549, DC, and 3T3 cells were treated with CaCe for 24 h. Cell death was assessed by annexin V and propidium iodide (PI) staining. The cells were detached, and cell pellets were incubated with annexin V‐Alexa Flour 488 (A13201; Thermo Fisher Scientific) for 15 min and PI (P1304MP, Thermo Fisher Scientific) for 15 min in the binding buffer (PBS, 1% bovine serum albumin [BSA]). The survival was estimated by population negative for annexin V and PI signals. Flow cytometry analysis was also used to detect GFP^+^ macrophages, and CD8^+^ T cells. The cell dissociates were incubated with CD8‐PE antibody (Clone SK1, BioLegend). Cell population profiles were detected using flow cytometry (BD FACSCalibur).

### Data analysis

Data were represented as mean and SD, which was analyzed by Student's *t* test, one/two‐way ANOVA analysis followed with corresponding post hoc test. Statistical significance was determined when *p* value was <0.05.

## RESULTS

### Characterization and cellular uptake of MDA‐MB‐231 derived exosomes

**FIGURE 1:**
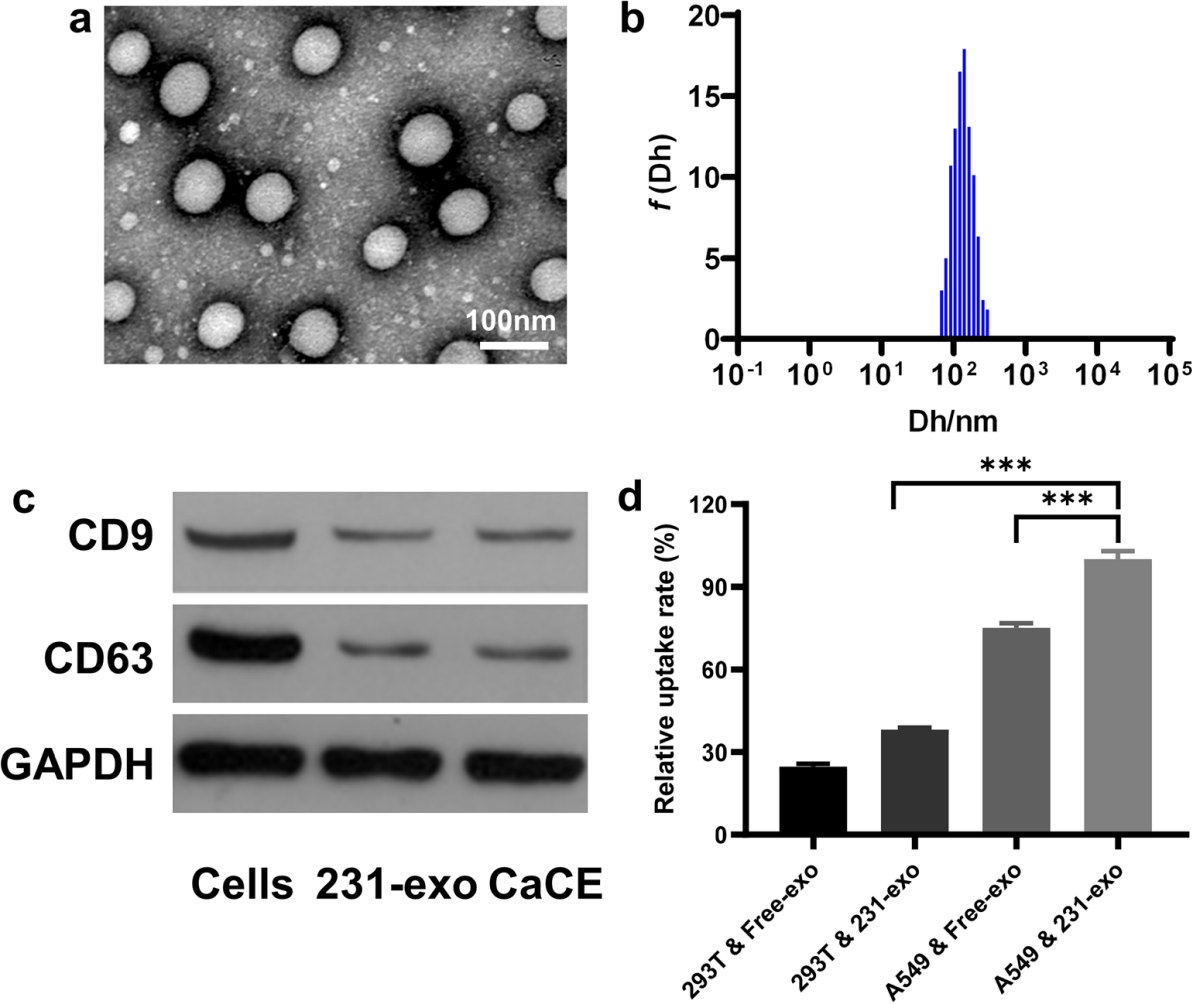
Characterization of cell‐derived exosome 231‐exo (CaCE). (a) Transmission electron microscopy (TEM) images of CaCE; (b) size distributions of CaCE; (c) 231‐exo and CaCE were analyzed by western blotting for the expression of the exosome‐specific markers CD9 and CD63. GAPDH was used as a loading. (d) Relative uptake rate of 293T and free‐exo, 293T and 231‐exo, A549 and free‐exo and A549 and 231‐exo using flow cytometry. Data were represented as mean ± SD. **p < 0.01; ***p < 0.001

Exosomes were isolated from the MDA‐MB‐231 breast cancer cell line (231‐exo). The transmission electron microscopy (TEM) imaging indicated the presence round vesicles of 50–100 nm in diameter with the typical exosome morphology, higher beam scattering on the edges, and decreased electron scattering in the center (Figure 1(a)). Dynamic light scattering quantification showed the size distribution of vesicles at around 100 nm (Figure 1(b)) consistent with TEM observations. The specificity of exosome isolate was confirmed by western blotting to exosome marker proteins. Lysates produced from the exosome isolate demonstrated strong positive bands for CD9 and CD63, confirming the exosome isolation and purity (Figure 1(c)).

The flow cytometry analysis indicated that relative uptake rate of 231‐exo in A549 cells significantly increased when compared to that in 293 cells (Figure 1(d)).

### Exosome engineering enhanced macrophage phagocytosis potential against LLC

**FIGURE 2:**
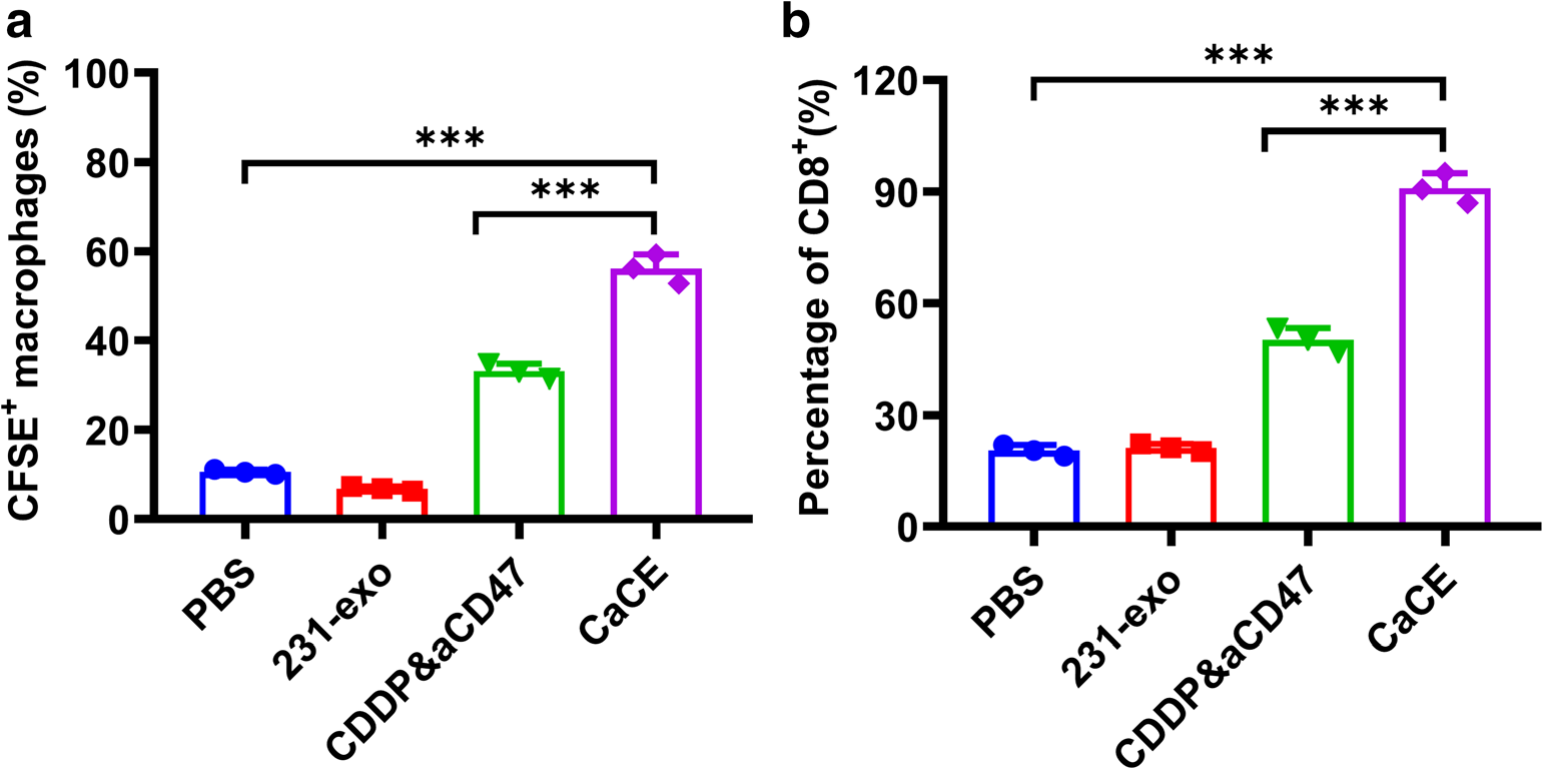
Cell‐derived exosome 231‐exo (CaCE) enhanced the phagocytic activity of macrophages for tumor cells. (a) The Lewis lung carcinoma (LLC) cells were co‐cultured with bone marrow flow cytometry derived macrophages (BMDMs). The cells were then harvested and stained with the F4/80^−^PE^−^Cy7 antibody. The percentages of F4/80^+^CFSE^+^ cells were then qualified. (b) BMDMs were co‐cultured with OVA‐expressing LLC cells and then incubated with CSFE^−^labeled CD8^+^ T cells. CFSE dilution assay was applied to determine the proliferation of CD8^+^ T cells. Data were represented as mean ± SD. **p < 0.01; ***p < 0.001. PBS, phosphate buffer saline

Macrophage phagocytosis serves as a conservative, crucial defensive mechanism against cancer development. In our study, we tested macrophage phagocytosis activity. LLC cells labeled with CFSE were co‐cultured with bone marrow‐derived macrophage cells. CSFE^+^/F4/80^+^ cell percentage were quantified. CDDP combined with anti‐CD47 treatment induced increased CFSE positive macrophages to ~30% (Figure 2(a)), suggestive of increased phagocytotic activity by combined treatment. This increase in phagocytosis of CFSE labeled LLC cell material was further increased in the CaCE treatment group, suggesting an advantageous effect of the exosome delivery system in improving macrophage phagocytotic response against cancer cells.

### CaCE increased T‐cell proliferation in response to LLC cell invasion

T‐cell proliferation serves as another crucial defense mechanism against cancer cell propagation. Here, we analyzed CD8^+^ T cell proliferation by CSFE dilution assay. CDDP pretreated LLC‐OVA cells were co‐cultured with BMDMs with or without CD47 antibody addition or CaCE addition. BMDM were then incubated with CSFE labeled CD8^+^ T cells in the presence or absence of CaCE. As shown in Figure 2(b), CD8^+^ (CFSE^+^) cell population increased to 53% in CDDP treatment with CD47 antibody, compared to 20% in the vehicle treatment group (PBS) and control 231‐exosomes treated groups. CaCE treatment further increased CD8^+^ cell to 90%, indicating exosome delivery of CD47 antibody enhanced the CD8^+^ T cell defensive mechanism compared to a CDDP and CD47 antibody combination.

### Delivery specificity of 231‐exo to A549 cancer cells

**FIGURE 3:**
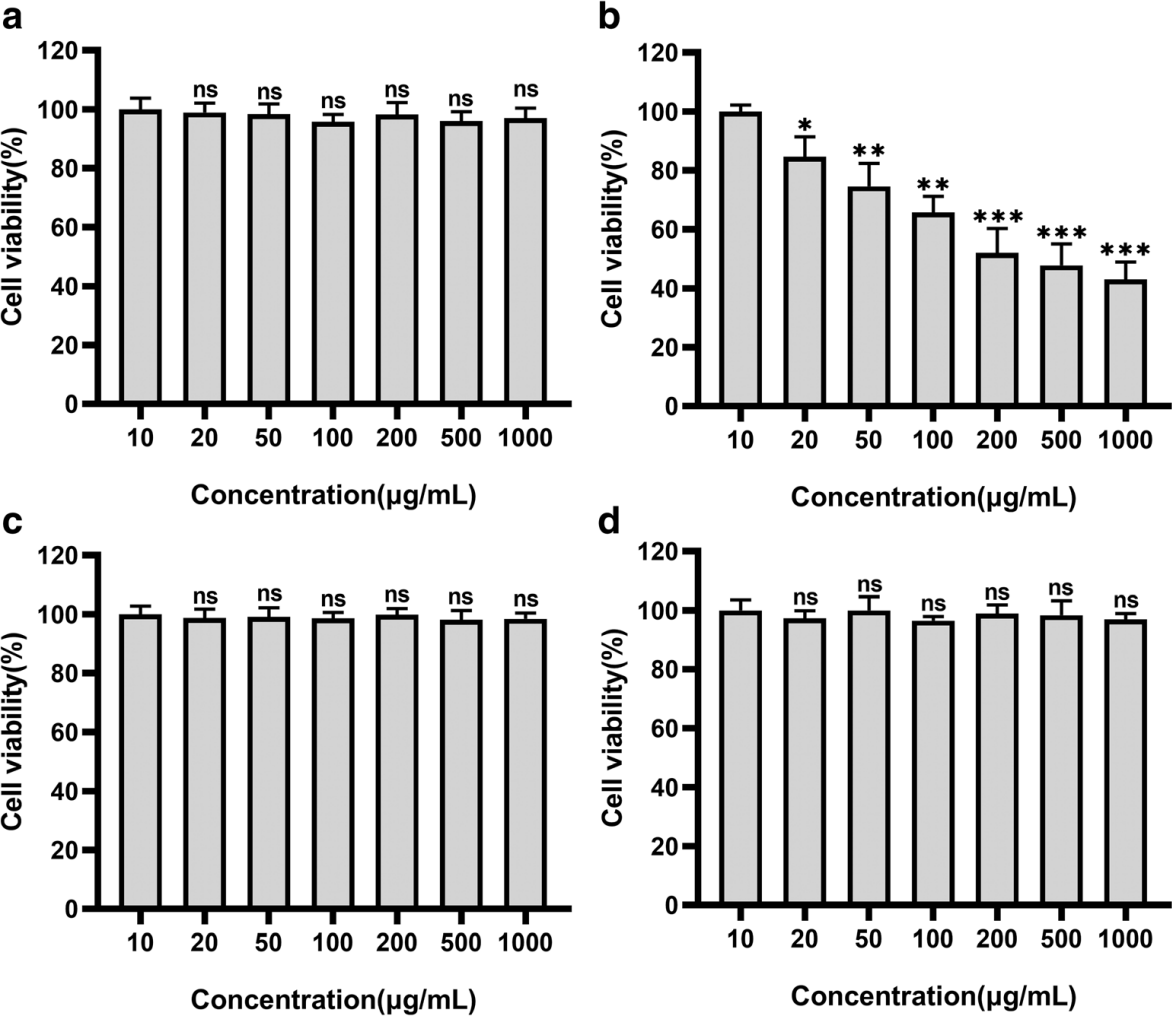
Effect of cell‐derived exosome 231‐exo (CaCE) in killing 293T and A549 cells in vitro. The cell viability of CaCE on 293T (a), A549 (b), DC (c), and 3T3 (d) cells treated with indicated concentrations of CaCE for 24 h. Data were expressed as mean ± SD, *n* = 6. **p < 0.01; ***p < 0.001

CaCE treatment in concentrations 10–1000 μg/mL have no toxic effects on 293T (Figure 3(a)), dendritic cells (Figure 3(c)) or NIH 3T3 cells (Figure 3(d)). However significant and the dose‐dependent cytotoxic effect was observed in the A549 cells (Figure 3(b)), suggestive of specific uptake of CaCE by the A549 cells, implying a therapeutic potential of CaCE in target cancer treatment.

### CaCE strongly suppressed tumor progression in LLC‐bearing mouse model

**FIGURE 4:**
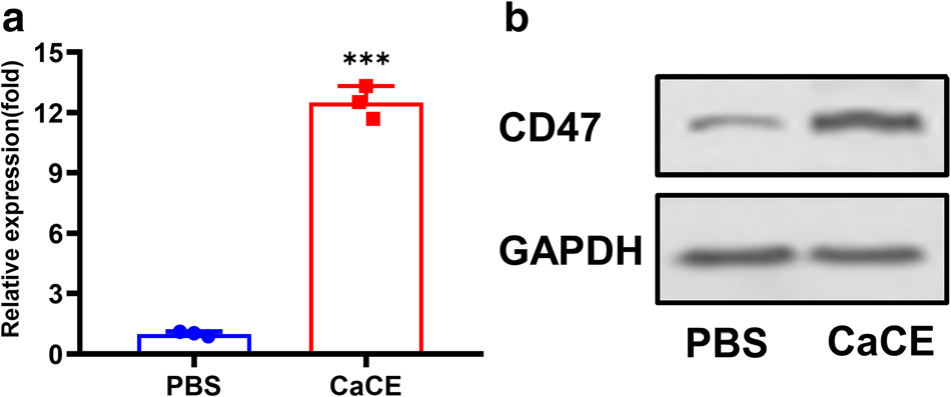
The antitumor effects of cell‐derived exosome 231‐exo (CaCE) on the Lewis lung carcinoma (LLC) tumor‐bearing mouse model. At the end of the experimental period, the mice were sacrificed, and the (a) messenger RNA (mRNA) expressions and (b) protein expression of CD47 in the tumor tissues were determined using real‐time polymerase chain reaction (PCR) and western blotting. Data were represented as mean ± SD. **p < 0.01; ***p < 0.001. PBS, phosphate buffer saline

After CaCE inoculation, we tested the expression level of CD47 in the tumor tissue. Exosomal delivery of anti‐CD47 induced the expression level of CD47 at both messenger RNA (mRNA) level (Figure 4(a)) and protein level (Figure 4b) compared to the vehicle control group.

**FIGURE 5:**
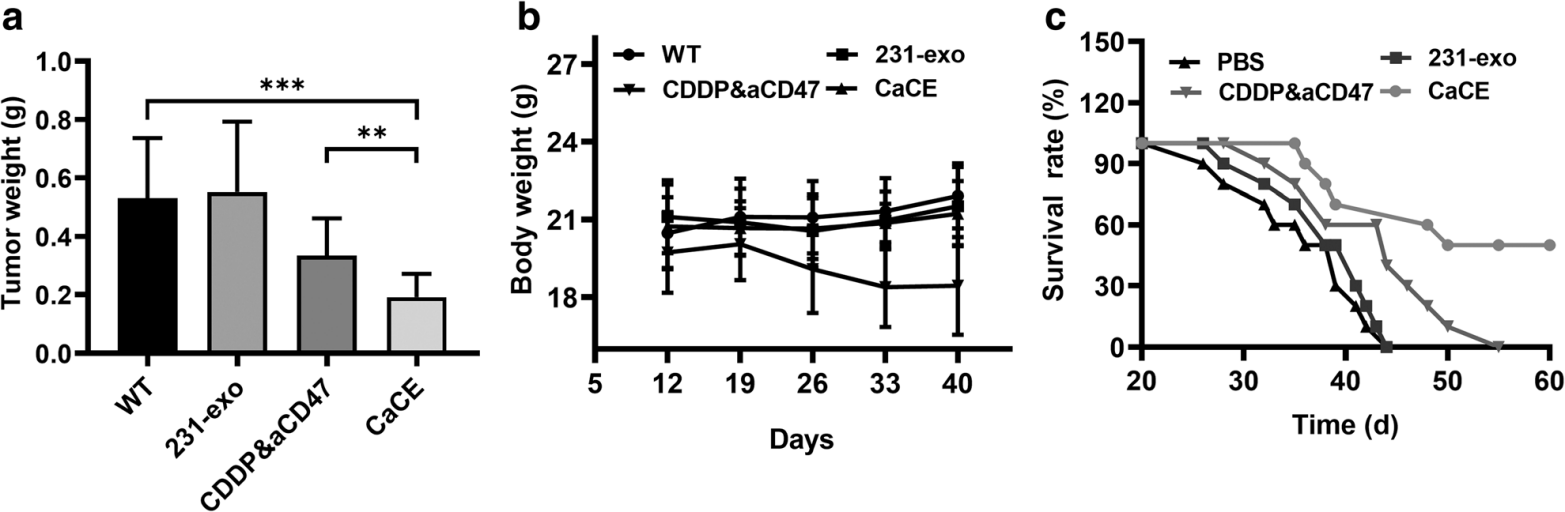
Antitumor efficacy of cell‐derived exosome 231‐exo (CaCE). The changes of (a) tumor tissue weight, (b) body weight, and (c) survival rates of the three experimental groups of tumor‐bearing mice during the 12‐day treatment. Data were expressed as mean ± SD, *n* = 8. **p < 0.01; ***p < 0.001

To compare the effect of exosome delivery of CDDP and aCD47 with the combined administration of CDDP and anti‐CD47 (CDDP&aCD47) on tumor formation, we monitored survival rate up to 60 days measured body weight tumor weight in LLC tumor‐bearing mouse model. Co‐administration of CDDP&aCD47 showed a tendency of decreased tumor weight, but it did not reach a significant difference (*p* > 0.05). Inoculation of the CDDP and anti‐CD47 exosomes showed dramatically decreased tumor weight compared to the vehicle treatment and co‐administration groups (Figure 5(a)). It was worth noting that the effects of synergistic treatment against tumor were more profound than either CDDP or aCD47 single treatment (Figure S1). No significant difference was observed in body weight change (Figure 5(b)). Consistently with tumor weight observations, the CaCE treatment group showed the highest survival rate, indicating a strong antitumor effect of the exosome facilitated the delivery of CCPD and CD47 antibodies (Figure 5(c)).

### CaCE applied its tumor‐suppressive effect by regulating the immune microenvironment in the LLC‐tumor‐bearing model

**FIGURE 6:**
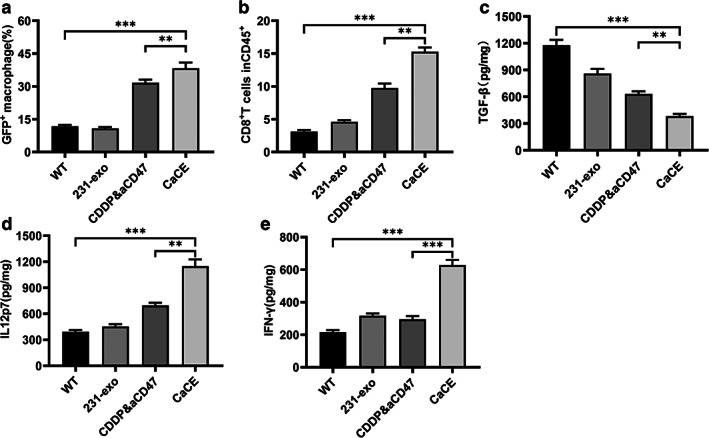
Treatment of cell‐derived exosome 231‐exo (CaCE) regulated the tumor immune microenvironment in the Lewis lung carcinoma (LLC) tumor‐bearing mouse model. (a) After 24 h of the last injection, the tumor tissues were collected, and the GFP signal in the F4/80 positive macrophages was determined using flow cytometry (*n* = 6). (b) The percentages of CD8^+^ T cells in the tumor tissues were also determined by using flow cytometry (*n* = 6). (c)−(e) The levels of cytokines including TGF‐β, IL‐12p70, and IFN‐γ in the tumor tissues were determined using specific enzyme‐linked immunoassay (ELISAs) (*n* = 8). Data were represented as mean ± SD. **p < 0.01; ***p < 0.001

We further explored the mechanism of CaCE anticancer effect by injection of GFP‐labeled LLC cells into wild‐type (WT) C57BL/6J mice, with the same four sets of treatment once the tumor size reached 50 mm^3^. Macrophage phagocytosis of LLC was analyzed by measuring the percentage of GFP^+^/F4/80^+^ cell population in tumor tissue with flow cytometry (Figure 6(a)). CDDP&aCD47 co‐administration tends to increase GFP^+^/F4/80^+^ population percentage. Exosome delivery of cisplatin and aCD47 dramatically increased GFP^+^ cells in F4/80^+^ population, indicating an enhancement of phagocytosis of LLC cells by macrophages in tumor tissue. Further flow cytometry analysis revealed a significant increase of CD8^+^ T cells in the CD45^+^ populations (Figure 6(b)). The increase in phagocytosis activity of macrophages implied the possible immune‐microenvironment change with the inoculation of CaCE. Indeed, we observed a significant increase in IL‐12p7 and IFN‐γ, two important cytokines for macrophage function, as well as the reduction in TGF‐β levels in tumor tissue (Figure 6(c),(d)). TGF‐β is well known to negatively affect T‐cell activation and phagocytotic capacity (Figure 6(e)).

## DISCUSSION

In the current study, we successfully engineered 231‐exo, an MBA‐DM‐231 breast cancer cell‐derived exosome with a commonly used chemotherapeutic drug, cisplatin and anti‐CD47 antibody. We examined the exosome co‐delivery system's anticancer effect both in vivo and in vitro. We observed an increased phagocytosis activity of BMDM co‐cultured with A549 cells with the addition of CaCE compared vehicle treatment group. Moreover, exosome delivery (CaCE) showed a significantly stronger effect than co‐administration of cisplatin and anti‐CD47 antibodies. In the LLC‐bearing lung carcinoma mouse model, CaCE administration significantly reduced tumor weight and increased survival rate, even compared to the co‐administration group. Further mechanism study showed an increase of IL‐12p7 and IFN‐γ with the administration of CaCE, suggesting that antagonizing CD47 improved resistance to cisplatin through immune microenvironment change.

Cisplatin is the first platinum‐based drug in chemotherapy to treat various cancers, including lung cancer.^25^ However, undesired drug resistance and side effects on multiple healthy tissues limited its clinical performance.^6^ In a previous study, we found that CD47 was increased with cisplatin treatment, whereas blocking CD47 with antibody improved the anticancer effect of cisplatin, implying the involvement of CD47 in cisplatin resistance.^14^ CD47 overexpression on multiple tumor cells inhibits phagocytosis of macrophages by interacting with signal regulatory protein α (SIRPα),^26^ initiating a “do not eat me” signal,^27^ therefore contributing to resistance to cisplatin treatment. The current study showed an even stronger phagocytotic effect with the exosome co‐delivery system compared to co‐administration of cisplatin and anti‐CD47. This is most likely because of the increased delivery efficiency of the exosome of CD47, neutralizing cisplatin treatment‐induced CD47 expression and resistance to cisplatin. Indeed, the CD47 expression level was increased in tumor tissues with CaCE treatment.

Exosomes play a crucial role in organotypic metastasis through multiple mechanisms, including membrane‐anchored integrin proteins, to induce inflammation, facilitating pre‐metastatic niches formation.^28^ This feature of exosomes from tumor cells provided a potential targeted drug delivery strategy.^29^ Indeed, a previous study by Nie et al.^22^ showed that 231‐exo could deliver miRNA‐126 specifically to non–small cell lung cancer. Integrinβ4 on the specific exosome interact with surfactant protein C on A549 cells, facilitating the tissue‐specific delivery cargos by 231‐exo to lung tumor cells. In our study, CaCE induced enhanced cell apoptosis in tumor tissue and phagocytosis by macrophage and improved survival rate, implying that exosome delivery targeted LLC developed from subcutaneous inoculation, rather than systemically attacking any tissue as in traditional chemotherapy. In the in vitro experiment with CaCE addition to cell cultures, only A549 cells responded to exosome‐delivered cisplatin treatment with increased apoptosis across several cell lines including A549cells, HEK293 cells, NIH3T3, and dendritic cells, further indicating its targeted therapeutic potential against lung cancer. CaCE treatment dramatically improved the survival rate of the LLC tumor‐bearing model, revealing reduced systemic toxicity of cisplatin with 231‐exo delivery likely because of the organotypic effect from exosome delivery.

## CONCLUSION

In summary, we successfully encapsulated and delivered cisplatin and anti‐CD47 antibody with 231‐exo. The exosome delivery system improved the survival rate in LLC tumor‐bearing mice compared to co‐administration of cisplatin and CD47 blocking antibodies. It was indicated that a dramatic improvement of phagocytosis effect delivering anti‐CD47 antibody improved cisplatin resistance from increased CD47 expression. Changes in cytokines IL12p, IFN‐γ, and TGF‐β levels in tumor tissues indicated a microenvironmental change, favoring T‐cell proliferation. Furthermore, CaCE specifically targeted A549 cells in vitro, indicating an improved selectivity of cisplatin treatment, probably because of an organotrophism of the exosome. Our study revealed the advantage of exosome co‐delivery of CD47 antibody with cisplatin over the drug co‐delivery system and provided crucial evidence for the potential application of 231‐exo in the treatment of lung tumors.

## CONFLICT OF INTEREST

The authors declare that they have no competing interests.

## Supporting information


**Figure S1** The changes tumor tissue weights of tumor‐bearing mice during different treatment for 12 days. Data were expressed as mean ± SD, *n* = 6. Data were represented as mean and SD. CE, 231‐exo with CDDP; aCE, 231‐exo with aCD47; **p < 0.01, ***p < 0.001. CaCE, cell‐derived exosome 231‐exo; CDDP, cisplatin
