# Supplementary material for: Lung‐specific exosomes for co‐delivery of CD47 blockade and cisplatin for the treatment of non–small cell lung cancer
Source: Thorac Cancer. 2022 Aug 27;13(19):2723–31. doi: 10.1111/1759-7714.14606 (PMC9527158; doi:10.1111/1759-7714.14606)
Supplement: Supplementary file 1 — Figure S1 The changes tumor tissue weights of tumor‐bearing mice during different treatment for 12 days. Data were expressed as mean ± SD, n = 6. Data were represented as mean and SD. CE, 231‐exo with CDDP; aCE, 231‐exo with aCD47; **p < 0.01, ***p < 0.001. CaCE, cell‐derived exosome 231‐exo; CDDP, cisplatin [file TCA-13-2723-s001.docx]

**Supplementary materials**


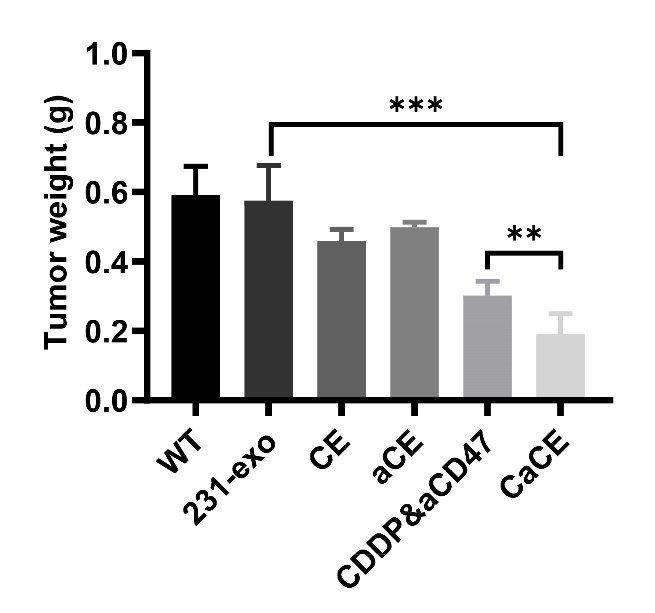


Figure S1. The changes tumor tissue weights of tumor-bearing mice during different treatment for 12 days. Data were expressed as mean ± SD, n = 6. Data were represented as mean and SD. CE: 231-exo with CDDP; aCE: 231-exo with aCD47; **p < 0.01, ***p < 0.001.
